# Family Health History Completeness in Prenatal Genetic Counseling: An Exploratory Study at a Single University Hospital

**DOI:** 10.3390/healthcare13172233

**Published:** 2025-09-06

**Authors:** Tomoharu Tokutomi, Akiko Yoshida, Kunihiko Miura

**Affiliations:** 1 Department of Pediatrics, Kawasaki Medical School, Kurashiki 701-0192, Japan; 2 Department of Clinical Genetics, School of Medicine, Iwate Medical University, Morioka 020-8505, Japan; akikoyos@iwate-med.ac.jp; 3 Iwate Prefectural Miyako Hospital, Miyako 027-0096, Japan; miurakun8@gmail.com

**Keywords:** FHH (family health history), genetic counseling, disease onset, patient education, data quality

## Abstract

**Background:** Family health history (FHH) is essential for genomic medicine and risk assessment, but its completeness in Japanese prenatal settings is poorly understood. Prior studies show that details such as cause of death (COD) and age at onset are often missing. To address this gap, we conducted a pilot observational exploratory study evaluating FHH completeness in a Japanese prenatal genetic counseling setting. **Methods:** We analyzed data from 24 participants (12 couples) who underwent prenatal genetic counseling at a university hospital, most of whom were of advanced maternal age and had undergone non-invasive prenatal testing (NIPT). FHH was collected using a structured form at the first visit and revised at the second visit. Completeness was assessed for four items: medical history, age at death, COD, and age at disease onset. Associations with participant characteristics were also explored. **Results:** Disease history was most complete, while COD and age at onset were frequently missing. Age at death was more complete than COD, indicating that information on deceased relatives or timelines was harder to obtain. Participants with personal or family medical conditions tended to provide more complete FHH. The structured form and opportunity for revision likely enhanced completeness. **Conclusions:** This pilot study shows that COD and age at onset are the least complete components of FHH in Japanese prenatal counseling. The small sample size and single-hospital setting limit the generalizability of the findings, but they suggest that structured prompts and preparation before visits may improve FHH completeness and enhance risk assessment in clinical practice.

## 1. Introduction

Family health history (FHH) refers to the current or past medical conditions of biologically related relatives and is widely recognized as a predictive tool for assessing disease risk. Historically, it has played a crucial role in diagnosing and evaluating the risk of monogenic diseases, as well as in considering patients’ psychosocial backgrounds during clinical care [1]. Since the early 2000s, FHH has also been utilized in risk stratification for common multifactorial diseases, such as cancer, cardiovascular disease, and type 2 diabetes [1], to guide surveillance, genetic testing, and genetic counseling [2,3,4]. For some hereditary conditions, including hereditary breast and ovarian cancer syndrome, Lynch syndrome, and familial hypercholesterolemia, FHH can serve as the sole risk predictor [5].

Recent prevention strategies, such as cascade screening for relatives of affected individuals and child–parent screening, further emphasize the role of FHH in primary care [6,7,8,9]. Accurate risk assessment requires comprehensive and up-to-date information on family history [10,11,12,13,14,15]. Essential elements include relatives’ ages, age and cause of death, disease names and age at diagnosis, family relationships, ethnic background, consanguinity, relevant negative findings, and environmental factors [2,10,11,12,13].

However, several barriers hinder the effective collection of FHH, including limited patient knowledge and time constraints in clinical practice [2,11,16]. For example, fewer than 4% of FHHs collected in primary care contain sufficient information to evaluate multifactorial disease risk [17]. Although a U.S. study showed that updating FHH data improves completeness and clinical utility [2], no study in Japan has examined the specific content of FHH in detail.

This study aimed to evaluate the completeness and quality of FHH collected in a Japanese primary care genetic counseling setting and to explore their relationship with participant characteristics, including sex, presence of personal medical conditions, and family size.

## 2. Materials and Methods

This study targeted adult participants in their 20s to 40s, commonly referred to as the “child-rearing generation”, who visited a genetic counseling clinic for prenatal diagnostic purposes. We calculated the completeness of information regarding family members and their diseases (i.e., FHH quality) for each pedigree and analyzed its relationship with participants’ individual characteristics.

### 2.1. Participants

This study was designed as an observational exploratory study, conducted at the Department of Clinical Genetics, Iwate Medical University Hospital, and its affiliated Uchimaru Medical Center. Although the study involved structured data collection at two time points, no experimental intervention was applied, and the design focused on identifying patterns in FHH quality within a small, intentionally selected sample. The study population consisted of pregnant individuals and their partners who visited the hospital for prenatal genetic diagnosis, including non-invasive prenatal testing (NIPT) or maternal serum marker screening. A Certified Genetic Counselor (Japan) or a study coordinator recruited genetic counseling clients for study participation. Individuals who provided written informed consent were enrolled in the study.

Participants were excluded if a Board-Certified Clinical Geneticist (Japan) determined that participation would pose an undue psychological burden due to abnormal pregnancy findings or test results. The study protocol was approved by the Ethics Committee of Iwate Medical University School of Medicine (approval number: H2019-005).

### 2.2. Study Procedure

FHH data were collected using a structured form and administered twice. Participants completed the form either while waiting for their initial counseling session or in advance by downloading it from the hospital website. After the first session, those who agreed to participate were provided with a copy of their completed FHH form and instructed to revise or update it before the second visit. The revised forms were collected during the follow-up session. Although FHH data were collected at two time points, the present analysis was conducted using only the final, revised version submitted during the second session. With the participants’ consent, attending physicians were permitted to supplement missing data during clinical interviews, such as unreported medical conditions of relatives. Thus, the study includes minor elements of a pre-post improvement design with supplemental input, though no formal intervention or educational program was provided between sessions. We did not conduct a paired comparison between the first and second versions; analyses focused exclusively on the final, clinician-reviewed forms.

FHH data were collected using a structured form and administered twice. Participants completed the form either while waiting for their initial counseling session or in advance by downloading it from the hospital website. After the first session, those who agreed to participate were provided with a copy of their completed FHH form and instructed to revise or update it before the second visit. The revised forms were collected during the follow-up session.

### 2.3. Family Health History Form

Participants were asked to document family health information for themselves and their biological relatives, including siblings, parents, children, nieces/nephews, uncles/aunts, and grandparents. The following attributes were recorded for each individual: birth order, sex, vital status (alive or deceased), age or age at death, medical history (including specific disease names), and COD (if applicable). The form allowed annotation of both disease or healthy status, and encouraged the use of approximate ages (e.g., “in their 50s”) when exact data were unavailable. Participants completed the form either during outpatient visits or at home.

### 2.4. Evaluation of Family Health History Quality

The FHH quality was assessed using the method proposed by Beadles et al. [2], focusing on the completeness of four key parameters for each pedigree:

1. Disease history completeness
  - Number of relatives (excluding the participant) with reported disease or confirmation of health status/Total number of relatives.
2. Age at death completeness
  - Number of deceased relatives with recorded age at death/Total number of deceased relatives.
3. COD completeness
  - Number of deceased relatives with documented COD/Total number of deceased relatives.
4. Age of disease onset completeness
  - Number of diseases with reported age of onset/Total number of diseases.

For items restricted to deceased relatives (age at death and COD), completeness was calculated separately for each participant, using only their deceased relatives as the denominator. These individual completeness values were then summarized across participants (median and range). Approximate age ranges (e.g., “in their 50s”) were accepted for age at death and age of onset. All calculations excluded the participants. Diseases were classified according to categories adapted from those used in the MeTree patient-facing pedigree tool [18]. Only the disease category framework was referenced; the MeTree platform itself was not implemented in this study. The categories included abdominal aortic aneurysm, hypertension, lupus, multiple sclerosis, obesity, osteoporosis, thyroid disorders, rheumatoid arthritis, coagulation disorders, cerebrovascular disease, cancer, cardiovascular disease, diabetes, gastrointestinal disease, eye disorders, hereditary cancer syndromes, hereditary cardiovascular syndromes, hyperlipidemia, renal disease, liver disease, respiratory disease, psychiatric disorders, and sickle cell disease/thalassemia. Two independent raters reviewed all FHH forms to assess quality parameters. No discrepancies were identified between raters; therefore, inter-rater reliability statistics were not calculated.

### 2.5. Statistical Analysis

Descriptive statistics were used to summarize participant characteristics. Comparisons among the four FHH quality parameters were conducted using the Wilcoxon signed-rank test with the Bonferroni correction. The influence of sex and personal medical conditions on FHH quality was assessed using the Wilcoxon rank-sum test. Additionally, Spearman’s rank correlation coefficients were calculated to assess the association between family size and FHH quality. For pairwise comparisons, Hodges–Lehmann estimates of the median difference with 95% confidence intervals (CI) were calculated from Wilcoxon tests. For correlations, Spearman’s rho was calculated with 95% confidence intervals. Statistical significance was set at a two-sided *p* < 0.05. All analyses were performed using f-tree (version 4.0.2) [19], Microsoft Excel 2016, and R software (version 3.5.1).

## 3. Results

### 3.1. Participant Characteristics (Table 1)

During the study period (18 June to 19 December 2019), 110 clients had prenatal testing. Among them, 92 individuals were from 46 NIPT cases (46 couples), and 18 were from 12 maternal serum marker cases (6 couples and 6 unaccompanied women).

Of the 110 clients, 30 received genetic counseling from the study physician or with the coordinator present. One individual was not recruited due to concerns about emotional burden, leaving twenty-nine invited to participate. Of those invited, one couple (2 individuals) declined due to work commitments, resulting in 27 initial consents. One couple (2 individuals) was excluded as their second visit was after the study period, so 25 provided consent and updated FHH forms within the period. Of the 25, 12 couples (24 individuals) participated together, while one woman attended alone without her partner. As her participation conditions differed from those of the couple-based framework, she was excluded from the analysis to ensure consistency and comparability across participants.

The final analytic sample consisted of 24 participants (12 couples), comprising 22 from the NIPT group (91.7%) and two from the maternal serum marker group (8.3%), with an equal number of males and females. No participants met the exclusion criteria.

The median age of all participants was 40 years (mean: 39.4; range: 32–46 years). For males, the median was 41 (mean: 40.2; range: 32–46), and for females, the median was 39 (mean: 38.6; range: 33–42). The median number of relatives per participant was 13 (mean, 14.2; range: 9–24), with 340 relatives reported across all pedigree classes.

The gestational age of the 12 pregnant women at the time of participation ranged from 11 to 15 weeks: two were at 11 weeks, four at 12 weeks, two at 13 weeks, three at 14 weeks, and one at 15 weeks.

### 3.2. Family Health History Quality (Table 2)

We evaluated the quality of FHH across the 24 participating pedigree groups using four parameters, each expressed as a measure of completeness: disease history, age at death, COD, and age at disease onset. The median completeness for each parameter was as follows: disease history completeness, 80.0% (mean, 76.6%; range, 33.3–100%); age at death completeness, 85.4% (mean, 62.9%; range, 0.0–100%); COD completeness, 25.0% (mean, 31.6%; range, 0.0–100%); and age at disease onset completeness, 0.0% (mean, 19.2%; range, 0.0–77.8%). Disease history completeness was significantly higher than both COD completeness (*p* < 0.001, 95% CI [28.0, 70.0]) and age at disease onset completeness (*p* < 0.001, 95% CI [41.4, 73.1]). Age at death completeness was also significantly higher than the COD completeness (*p* = 0.015, 95% CI [33.3, 87.5]) and age at disease onset completeness (*p* = 0.001, 95% CI [38.9, 80.1]).

### 3.3. Sex-Based Comparison of Family Health History Quality (Table 3)

We conducted a sex-based analysis of FHH quality across four parameters: disease history completeness: male, median 80.0%, mean 72.1% (range: 33.3–100%); female, median 77.0%, mean 81.1% (range: 58.8–100%), age at death completeness: male, median 81.7%, mean 56.3% (range: 0.0–100%); female, median 100%, mean 69.4% (range: 0.0–100%), COD completeness: male, median 0.0%, mean 24.3% (range: 0.0–100%); female, median 29.2%, mean 38.9% (range: 0.0–100%), and age of disease onset completeness: male, median 0.0%, mean 18.3% (range: 0.0–55.6%); female, median 0.0%, mean 19.9% (range: 0.0–77.8%). Although both the median and mean values tended to be similar or higher in female across all parameters, no statistically significant differences were observed between males and females in disease history completeness (*p* = 0.449, 95% CI [−28.3, 10.6]), age at death completeness (*p* = 0.306, 95% CI [−50.0, 25.0]), COD completeness (*p* = 0.298, 95% CI [−50.0, 8.3]), or age at disease onset completeness (*p* = 0.906, 95% CI [−20.0, 17.2]).

### 3.4. Disease History and Its Impact on Family Health History Quality (Table 4, Table 5 and Table 6)

We first summarized the diseases reported by the participants and their family members (Table 4 and Table 5). Among the 24 participants, four individuals (16.7%) reported having a disease that fell within the predefined categories: diabetes, psychiatric disorder, gastrointestinal disease, and thyroid disease (one participant each). In addition, four individuals reported other medical conditions, including chronic sinusitis, allergic rhinitis, uterine fibroids, ovarian endometriotic cysts, atopic dermatitis, and asthma. Seven participants (29.1%) were classified as having a disease, with one individual reporting two conditions (*n* = 8; total disease entries).

Among the 340 relatives included in the pedigree, the most frequently reported self-disclosed conditions were cancer (30 individuals, 8.8%), diabetes and hypertension (14 each, 4.1%), cerebrovascular disease (12, 3.5%), cardiovascular disease (9, 2.6%), and psychiatric disorders (8, 2.4%). Next, we compared FHH quality across the four parameters between participants with and without a disease (Table 6). Participants with a disease (*n* = 7) demonstrated significantly higher FHH quality than those without a disease (*n* = 17) in three of the four parameters: disease history completeness (88.9% vs. 70.0%; mean: 89.9% vs. 71.2%, *p* = 0.026, 95% CI [0.0, 31.9]); COD completeness (50.0% vs. 0.0%; mean: 56.0% vs. 21.6%, *p* = 0.038, 95% CI [0.0, 83.3]); and age of disease onset completeness (53.8% vs. 0.0%; mean: 39.2% vs. 9.9%, *p* = 0.019, 95% CI [0.0–55.6]). No significant difference in age at death completeness was observed between the two groups (median: 100.0% vs. 80.0%, *p* = 0.377, 95% CI [−4.2, 75.0]). These results suggest that participants with diseases may be more aware of their FHH, which could lead to more complete reporting.

### 3.5. Impact of Family Size on Family Health History Quality (Figure 1)

**Figure 1:**
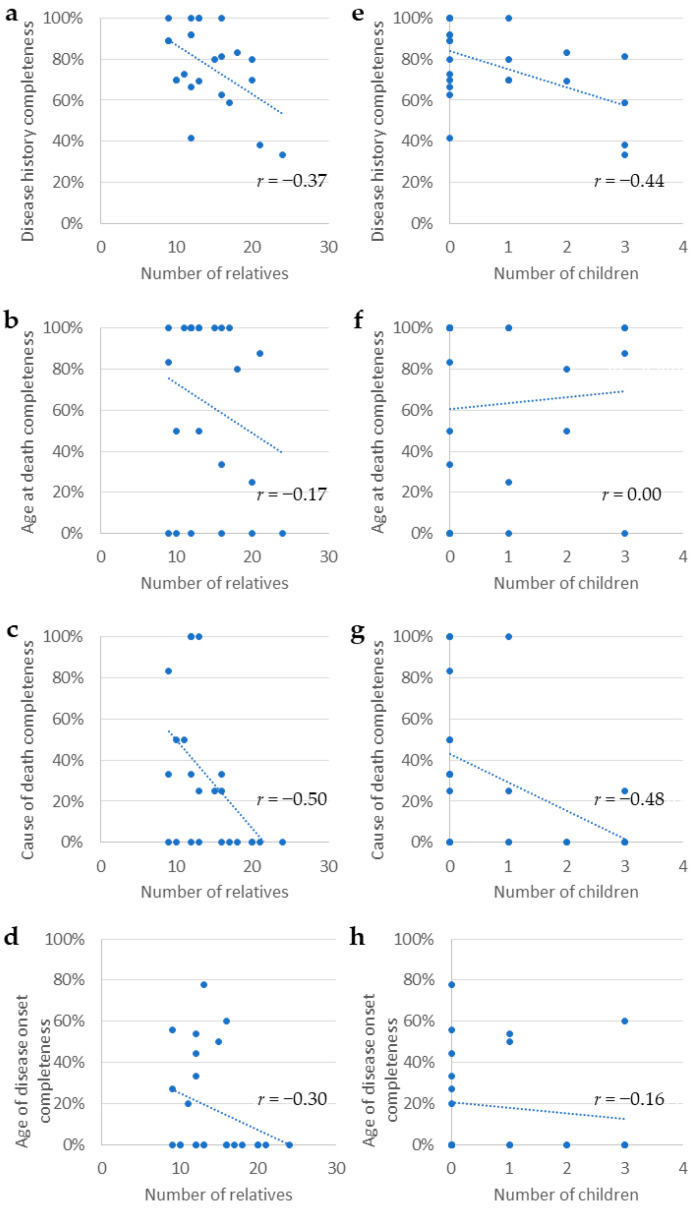
Correlation between family composition and family health history quality (*N* = 24). All coefficients were calculated using Spearman’s rank correlation. 95% confidence intervals are reported in the main text. Correlation coefficients with moderate or stronger effect sizes were defined as |*r*| ≥ 0.4 (Cohen’s convention). Panels show: (**a**) number of relatives vs. disease history completeness; (**b**) number of relatives vs. age at death completeness; (**c**) number of relatives vs. cause of death completeness; (**d**) number of relatives vs. age at disease onset completeness; (**e**) number of children vs. disease history completeness; (**f**) number of children vs. age at death completeness; (**g**) number of children vs. cause of death completeness; (**h**) number of children vs. age at disease onset completeness. Two missing values were excluded from the age of disease onset completeness (**d**,**h**).

The correlation between family size and FHH quality was examined using four parameters. The number of relatives (excluding the participant) was negatively correlated with COD completeness (*r* = −0.50, 95% CI [−0.785, −0.117], Figure 1c). In contrast, no significant correlations were observed for the other three parameters (disease history completeness: −0.37, 95% CI [−0.693, 0.079], Figure 1a, age at death completeness: −0.17, 95% CI [−0.593, 0.278], Figure 1b, and age of disease onset completeness: −0.30, 95% CI [−0.641, 0.132], Figure 1d).

Additionally, the number of participants’ children was negatively correlated with disease history completeness (*r* = −0.44, 95% CI [−0.734, −0.048], Figure 1e) and COD completeness (*r* = −0.48, 95% CI [−0.732, −0.125], Figure 1g), with no significant correlation observed for age at death completeness (*r* = 0.00, 95% CI [−0.396, 0.394], Figure 1f) or age at disease onset completeness (*r* = −0.16, 95% CI [−0.557, 0.317], Figure 1h).

## 4. Discussion

This pilot observational exploratory study evaluated the completeness of family health history (FHH) collected in a prenatal genetic counseling setting at a single university hospital and examined its relationship with participant characteristics. This study has several limitations that should be taken into account when interpreting the results. First, the small sample size limits statistical power and the generalizability of our findings. Although trends were consistent with our hypotheses for sex and personal medical conditions, these results should be interpreted cautiously, as confidence intervals often included values compatible with no association. Second, our participants were recruited from a single university hospital, which may have attracted individuals with a greater baseline interest or knowledge about health and family history, potentially overestimating the completeness of our results compared with the general population. Third, FHH data were self-reported and not verified against medical records, which could introduce recall bias or inaccuracies. Fourth, the cross-sectional design precludes assessment of changes in completeness over time or the impact of interventions. Previous studies have also underscored that incomplete or inaccurate FHH can limit its usefulness for clinical risk assessment and preventive strategies [17,20,21].

Overall, disease history completeness exceeded completeness for cause of death (COD) and age at disease onset, and age at death completeness exceeded COD completeness. These findings suggest that items requiring knowledge of deceased relatives or specific timelines (e.g., age at onset) are more difficult for patients to report with precision than the presence/absence of disease.

Our results align with prior work, which shows that periodic updating of FHH improves data completeness and clinical utility [2]. In primary care settings, patient time constraints and limited knowledge about relatives remain significant barriers [2,11,16,17]. In this cohort, the routine use of a structured form and the opportunity to revise information before the second visit likely contributed to higher overall completeness. However, items dependent on information from multiple family members—particularly COD and age at onset—showed lower completeness, underscoring the need for targeted prompts and follow-up questioning.

We hypothesized, based on prior findings [11], that women, participants with personal or family medical conditions, and those with smaller families would provide more complete FHH. While trends were consistent with some expectations, the small sample size limited statistical power, and confidence intervals often included values compatible with no association, warranting cautious interpretation.

From a practical standpoint, these results highlight two priorities for improving FHH quality in similar settings. First, clinicians should proactively elicit COD and age-at-onset data using brief, standardized prompts (e.g., “Do you know what your grandparent died of?”/”About how old were they when symptoms began?”). Second, combining pre-visit forms with a brief, structured review during counseling can improve completeness without placing excessive burden on patients or providers. For patients anticipating prenatal testing, explaining the clinical importance of COD and the age at onset may further motivate them to gather information. More broadly, strengthening the FHH collection has the potential to enhance risk assessment, guide preventive care, and support patient engagement in genomic medicine, as highlighted in previous reports [22,23].

Despite its limitations, this study is the first to detail the completeness of FHH in a Japanese prenatal genetic counseling context, providing a foundation for targeted interventions to improve COD and age-at-onset reporting in clinical practice.

## 5. Conclusions

This exploratory study found that family health histories in Japanese prenatal genetic counseling rarely included the cause of death or age at disease onset. While the small sample size and single-site design limit generalizability, our findings suggest that structured prompts and encouraging patients to prepare family information in advance may help improve the completeness of family health histories. Such approaches could strengthen risk assessment and contribute to enhancing the quality of prenatal care.

## Figures and Tables

**Table 1:** Participant characteristics (*N* = 24).

1. Test Type: *n* (%)		
	Non-invasive prenatal testing (NIPT)	22 (91.7)
	Maternal serum marker screening	2 (8.3)
2. Sex: *n* (%)		
	Male	12 (50.0)
	Female	12 (50.0)
3. Age (years): Median, Mean, Range		
	Male	41, 40.2, 32–46
	Female	39, 38.6, 33–42
	Total	40, 39.4, 32–46
4. Number of relatives per participant (excluding self)		
	Median	13
	Mean	14.2
	Range	9–24
	Total number of relatives	340
5. Number of children: *n* (%)		
	0	14 (58.3)
	1	4 (16.7)
	2	2 (8.3)
	3	4 (16.7)
6. Gestational age at time of visit (weeks): number of pregnant women		
	11 weeks	2
	12 weeks	4
	13 weeks	2
	14 weeks	3
	15 weeks	1

*n*: number of participants; %: percentage.

**Table 2:** Family health history quality (*N* = 24).

Quality Parameters	Median	Mean	Range	Statistical Comparison (*p*-Value, Median Difference, 95% CI)
Disease history completeness	80.0	76.6	33.3–100.0	vs. Cause of death: <0.001, 49.6, 28.0–70.0
				vs. Age of disease onset ^†^: <0.001, 57.7, 41.4–73.1
Age at death completeness	85.4	62.9	0.0–100	vs. Cause of death: 0.015, 62.5, 33.3–87.5
				vs. Age of disease onset ^†^: 0.001, 61.1, 38.9–80.1
Cause of death completeness	25.0	31.6	0.0–100.0	N/A
Age of disease onset completeness ^†^	0.0	19.2	0.0–77.8	N/A

CI: Confidence interval, N/A: not applicable. All values in median, mean, and range columns are expressed as percentages. Two-sided *p*-values were calculated using the Wilcoxon signed-rank test with Bonferroni correction. Median differences and 95% confidence intervals (for an overall α = 0.05, the per-contrast level was set to 99.17%) were obtained using the Hodges–Lehmann estimator. Percentages for age at death and cause of death completeness were calculated for each participant using only their deceased relatives as the denominator. ^†^ Excluded two missing values.

**Table 3:** Sex-based comparison of family health history quality (*N* = 24).

Quality Parameters	Male (*n* = 12)			Female (*n* = 12)			*p*-Value, Median Difference, 95% CI
	Median	Mean	Range	Median	Mean	Range	
Disease history completeness	80.0	72.1	33.3–100.0	77.0	81.1	58.8–100.0	0.449, −7.2, −28.3–10.6
Age at death completeness	81.7	56.3	0.0–100.0	100.0	69.4	0.0–100.0	0.306, −4.2, −50.0–25.0
Cause of death completeness	0.0	24.3	0.0–100.0	29.2	38.9	0.0–100.0	0.298, −4.2, −50.0–8.3
Age of disease onset completeness ^†^	0.0	18.3	0.0–55.6	0.0	19.9	0.0–77.8	0.906, −1.9, −20.0–17.2

CI: Confidence interval. All values in median, mean, and range columns are expressed as percentages. Two-sided *p*-values were calculated using the exact Wilcoxon rank-sum test. Median differences (Male−Female) and their 95% confidence intervals were estimated using the Hodges–Lehmann estimator. Percentages for age at death and cause of death completeness were calculated for each participant using only their deceased relatives as the denominator. ^†^ Excluded two missing values from male.

**Table 4:** Self-reported disease history among participants (*N* = 24).

Disease Category	Number of Individuals (%)
Diabetes	1 (4.2)
Psychiatric disorder	1 (4.2)
Gastrointestinal disease	1 (4.2)
Thyroid disease	1 (4.2)
Other conditions *	4 (16.7)
Total ^†^	7 (29.1)

* Other conditions include chronic sinusitis, allergic rhinitis, uterine fibroids, ovarian endometriotic cysts, atopic dermatitis, and asthma; some participants reported multiple conditions. ^†^ One participant reported two conditions; total entries = 8, individuals = 7.

**Table 5:** Frequently reported diseases among relatives (*N* = 340).

Disease Category	Number of Individuals (%)
Cancer	30 (8.8)
Diabetes	14 (4.1)
Hypertension	14 (4.1)
Cerebrovascular disease	12 (3.5)
Cardiovascular disease	9 (2.6)
Psychiatric disorder	8 (2.4)

Less frequently reported conditions (e.g., osteoporosis, allergy-related conditions, gynecological disorders) were excluded from this summary table for clarity.

**Table 6:** Family health history quality by presence of participant disease (*N* = 24).

Quality Parameters	With a Disease (*n* = 7)			Without a Disease (*n* = 17)			*p*-Value, Median Difference, 95% CI
	Median	Mean	Range	Median	Mean	Range	
Disease history completeness	88.9	89.9	70.0–100.0	70.0	71.2	33.3–100.0	0.026, 18.8, 0.0–31.9
Age at death completeness	100.0	76.2	0.0–100.0	80.0	57.4	0.0–100.0	0.377, 1.7, −4.2–75.0
Cause of death completeness	50.0	56.0	0.0–100.0	0.0	21.6	0.0–100.0	0.038, 29.2, 0.0–83.3
Age of disease onset completeness ^†^	53.8	39.2	0.0–77.8	0.0	9.9	0.0–50.0	0.019, 27.5, 0.0–55.6

CI: Confidence interval. All values in median, mean, and range columns are expressed as percentages. Two-sided *p*-values were calculated using the exact Wilcoxon rank-sum test. Median differences (With a disease−Without a disease) and their 95% confidence intervals were estimated using the Hodges–Lehmann estimator. Percentages for age at death and cause of death completeness were calculated for each participant using only their deceased relatives as the denominator. ^†^ Excluded two missing values from the group of “without a disease”.

## Data Availability

The datasets include sensitive personal health information. The datasets generated and analyzed in the current study are available from the corresponding author upon reasonable request.

## Abbreviations

The following abbreviations are used in this manuscript:

FHH	Family Health History
NIPT	Non-Invasive Prenatal Testing
COD	Cause of death
